# Case Report: *Streptococcus gordonii* triggering suppurative parotitis and sepsis in a patient with Sjögren syndrome

**DOI:** 10.3389/fmed.2025.1667223

**Published:** 2025-11-18

**Authors:** Ye Wang, Lin-Feng Gong, Ze-Kun Wang, Hong-Bo Qian, Xiao-Qing Wu

**Affiliations:** 1 Department of Infectious Diseases, Xi’an Eighth Hospital, Shaanxi Provincial Hospital of Infectious Diseases, Xi’an, Shaanxi, China; 2 Clinical Medicine, Sun Yat-sen University, Guangzhou, Guangdong, China; 3 Department of Radiology, Xi’an Eighth Hospital, Shaanxi Provincial Hospital of Infectious Diseases, Xi’an, Shaanxi, China; 4 Clinical Laboratory Center, Xi’an Eighth Hospital, Shaanxi Provincial Hospital of Infectious Diseases, Xi’an, Shaanxi, China; 5 Department of Integrative Medicine, Xi’an Eighth Hospital, Shaanxi Provincial Hospital of Infectious Diseases, Xi’an, Shaanxi, China

**Keywords:** *Streptococcus gordonii*, Sjögren syndrome, suppurative parotitis, sepsis, case report

## Abstract

**Background:**

Acute suppurative parotitis is common in primary Sjögren syndrome due to parotid gland dysfunction. *Streptococcus gordonii* is an aggressive pathogen but is rarely reported in acute suppurative parotitis.

**Case summary:**

A 64-year-old woman with primary Sjögren syndrome, history of periodontitis, and recurrent parotid swelling developed acute suppurative parotitis from *Streptococcus gordonii*, quickly leading to sepsis. The pathogen was confirmed via pus and blood cultures, and effective antimicrobial treatment was initiated promptly, controlling the condition.

**Conclusion:**

This case highlights the rapid progression of *Streptococcus gordonii*-induced acute suppurative parotitis to sepsis, emphasizing the importance of primary Sjögren syndrome screening and managing periodontal infections in patients with recurrent parotid swelling.

## Introduction

Primary Sjögren syndrome (pSS) is a chronic autoimmune disorder that mainly affects exocrine glands, particularly salivary and lacrimal glands. The resultant dysfunction leads to multiple symptoms, including dry mouth and dry eyes (1). While non-infectious parotid gland enlargement is common in pSS, secondary infections, including bacterial parotitis, can occur due to decreased salivary flow and changes in the oral microbiota (2). *Streptococcus gordonii* (*S. gordonii*) is a commensal, opportunistic Gram-positive bacterium mediating host cell adhesion, biofilm formation, and excessive inflammatory responses, playing a key role in the pathogenesis of apical periodontitis and infective endocarditis (3). This case report discusses a rare instance of *S. gordonii*-induced parotitis and sepsis in a patient with pSS, underlining diagnostic and therapeutic challenges.

## Case presentation

The patient, a 64-year-old female (height 151 cm, body weight 49 kg, and body mass index 21.5 kg/m^2^), presented with a 7-h history of acute swelling and pain beneath the right earlobe. This episode was accompanied by a high fever (39.2 °C), chills, and progressively worsening swelling extending to the right side of her face and neck. A physical examination revealed conspicuous enlargement of the right parotid gland with facial and neck edema. This patient exhibited a 30-year history of dry eyes and mouth, requiring frequent use of eye drops and water to help swallow dry food. Additionally, she had untreated dental caries and calculus for many years. No accompanying symptoms, such as fatigue, lymphadenopathy, skin rash, or arthralgia, were observed. The patient exhibited no history of underlying systemic or infectious diseases, medication use, trauma, invasive dental procedures, smoking or alcohol consumption, or relevant family history of genetic diseases.

**FIGURE 1:**
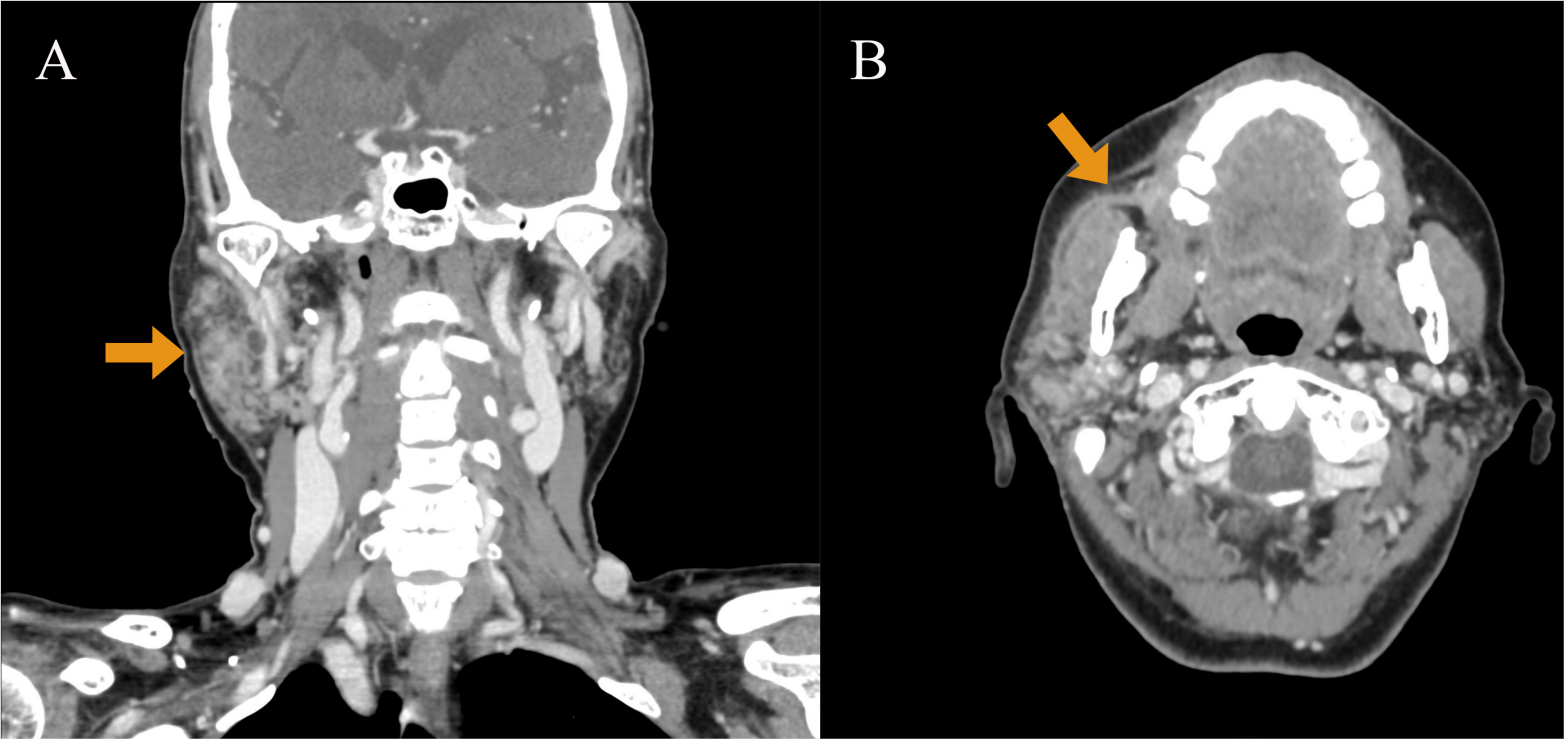
Enhanced computed tomography (CT) performed on July 15, 2022. **(A)** CT enhanced coronal image: it can be observed that the right parotid gland is significantly enlarged compared with the contralateral side (indicated by arrows). Thick-walled low-signal shadows can be observed inside, which are ring-shaped enhancements, involving the right orifice. **(B)** Enhanced CT axial image: the right parotid gland duct has fluid accumulation, a thickened and rough wall, and extremely conspicuous duct-like enhancement (indicated by arrows).

Initial laboratory investigations suggested elevated inflammatory markers: White blood cell count (WBC) 12.22 × 10^9^/L, neutrophil percentage (NE%) 81.85%, amylase 145.8 U/L, procalcitonin 4.5 ng/mL, and lactic acid 2.87 mmol/L. Ultrasound examination revealed inflammation in the right parotid and submandibular glands. Enhanced computed tomography (CT) revealed extensive cellulitis around the right parotid gland, extending into the deep neck spaces, encompassing the parapharyngeal and retropharyngeal spaces (Figure 1). The infection also involved right masseter and platysma muscles, with compression of the oropharyngeal structures. Based on these findings, she was diagnosed with suppurative parotitis. Empirical treatment with intravenous penicillin (1.6 million units/6 h) was initiated, along with local drainage and supportive care.

However, the patient constantly suffered from high fever (up to 39.8 °C) and higher leukocytosis (WBC: 19.62 × 10^9^/L). Procalcitonin levels were dramatically heightened (10.70 ng/mL), suggesting severe bacterial infection. Autoimmune disease antibodies revealed positive anti-SSA, antinuclear, antimitochondrial, and anti-Ro 52 antibodies. Corneal staining and tear secretion tests were both positive. Blood and pus cultures were positive for *S. gordonii* (sensitive to penicillin, erythromycin, ceftriaxone, levofloxacin, vancomycin, linezolid, and meropenem).

On the fourth day of hospitalization, following diagnosis of *S. gordonii* infection, sepsis, and pSS, the penicillin dose was increased to 4 million units every 6 h. The patient’s fever subsided, parotid gland swelling began to reduce, and pain decreased. Blood and pus cultures from the parotid duct were returned negative. She was discharged on day 23 with mild residual parotid swelling but no signs of active infection. At follow-up 11 days post-discharge, parotid swelling had completely resolved, with no further discharge from the parotid duct.

The patient has been followed up for 2 years. After discharge, she was referred to the rheumatology department; however, no immunomodulatory therapy, including hydroxychloroquine, was initiated for pSS. In October 2022, she underwent root canal treatment for pulpitis at a local dental hospital. Following this period and the initial periodontal treatment, she was lost to further specialist follow-up and did not undergo any subsequent laboratory or imaging monitoring. In July 2024, right parotid gland swelling and pain returned. Although suppurative parotitis has not recurred post-periodontal treatment, her pSS and recurrent parotid enlargement remained unmitigated.

## Discussion

In general, pSS is characterized by chronic inflammation of exocrine glands, particularly the salivary and lacrimal glands, resulting in xerostomia and keratoconjunctivitis sicca (2). Parotid gland enlargement occurs in up to one-third of patients with pSS and may be intermittent or persistent (4). This patient meets the 2016 American College of Rheumatology/European League Against Rheumatism classification criteria for pSS, exhibiting daily xerostomia for >3 months, seropositivity for anti-SSA antibodies, and an objective ophthalmological evaluation confirming a positive corneal staining test in conjunction with abnormal tear secretion (5). The patient declined to undergo a labial gland biopsy due to its invasive nature. Notably, bacterial infections, including suppurative parotitis, occur more frequently in patients with pSS due to lowered salivary flow and oral microbiome alteration (3, 6). Simultaneously, this patient presented with dental caries but had no history of tooth extraction or oral trauma.

*Streptococcus gordonii* has been implicated in various systemic infections (7, 8), including endocarditis (9–12), brain abscesses (9), septic arthritis (11, 13, 14), finger infections (15), empyema (16), and septic shock (17). *S. gordonii* exhibits unique virulence factors that enhance its invasiveness and can lead to severe infections. In patients with pSS, the markedly diminished salivary flow (unstimulated flow rate < 0.1 mL/min) compromises oral clearance, facilitating the bacterium’s transition from commensal to an invasive pathogen (1). Salivary fibronectin facilitates the adherence of both streptococcal and staphylococcal species, thereby giving rise to infection (18). Simultaneously, untreated periodontal disease of this patient created a persistent biofilm nidus with fibronectin secretion, further promoting bacterial adhesion to parotid duct epithelium (3). The interaction between host immune dysregulation and bacterial virulence might have contributed to the rapid progression of the disease in this case. Empirical therapy with penicillin was initiated to target typical pathogens of suppurative parotitis but was ineffective for this patient; however, increasing the dosage successfully controlled the infection. This outcome may be attributed to the protective effects of the biofilm barrier (19, 20). Based on the susceptibility results and in consideration of the potential biofilm effect, the dosage was adjusted to 4 million units every 6 h, successfully controlling the infection.

**FIGURE 2:**
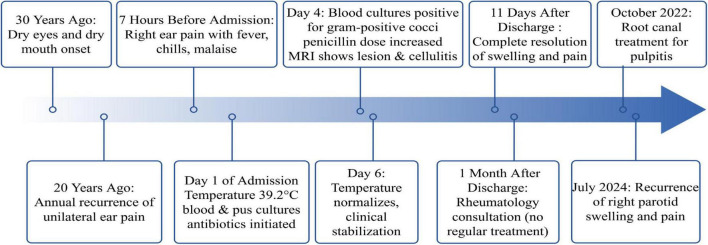
Timeline of clinical events in a patient with *Streptococcus gordonii* suppurative parotitis and sepsis.

The patient’s chronic, untreated dental caries possibly served as functioned as a continuous microbial reservoir, consistent with literature establishing poor oral hygiene as a risk factor for suppurative parotitis (21, 22). Within the context of pSS-related salivary hypofunction, this condition created an optimal environment for ascending infections through the ductal system, highlighting the essential role of rigorous periodontal care in this patient demographic. Post-discharge periodontal treatment effectively prevented parotitis recurrence for 2 years, emphasizing the importance of early pSS diagnosis and periodontal management. This timeline (Figure 2) outlines the patient’s journey from prolonged neglect of dental caries and recurrent parotid swelling—which established a susceptible host—to the aggressive infection by *S. gordonii* that culminated in sepsis. The patient expressed that the sudden, severe infection was a frightening experience, that highlightinged the importance of her previously neglected dental health.

This study’s limitations include the lack of periodontal pathogen profiling and insufficient systemic follow-up, which might have contributed to the recurrence. These findings emphasize the need to establish a standardized management protocol for patients with pSS and periodontal involvement, which warrants regular oral microbial screening and lifelong periodontal maintenance therapy.

## Conclusion

This case emphasizes the critical need for systematic screening for pSS in patients with suppurative parotitis, alongside the prompt and targeted identification of pathogens, including *S. gordonii*, to enhance clinical management. Besides, it underscores the importance of proactive oral healthcare in individuals with autoimmune disorders to reduce the risk of secondary infections.

## Data Availability

The original contributions presented in this study are included in this article/supplementary material, further inquiries can be directed to the corresponding author.
